# Gastric tumor mimicking bronchial tissue associated with a laryngotracheoesophageal cleft: a case report

**DOI:** 10.1186/s40792-023-01650-7

**Published:** 2023-05-09

**Authors:** Erika Nakatani, Keita Terui, Mitsuyuki Nakata, Shugo Komatsu, Ryohei Shibata, Satoru Oita, Yunosuke Kawaguchi, Ayako Takenouchi, Sakurako Harada-Kagitani, Takashi Kishimoto, Koji Fukumoto, Tomoro Hishiki

**Affiliations:** 1 Department of Pediatric Surgery, Chiba University Hospital, 1-8-1 Inohana, Chuo-ku, Chiba, 260-8670 Japan; 2 Department of Molecular Pathology, Chiba University Graduate School of Medicine, Chiba, Japan; 3 Department of Pediatric Surgery, Shizuoka Children’s Hospital, Shizuoka, Japan; 4 Department of Surgery, Japanese Red Cross Narita Hospital, Narita, Japan

**Keywords:** Laryngotracheoesophageal cleft, Gastric tumor, Bronchial tissue, Abnormal, Foregut cysts, Bronchopulmonary foregut malformation

## Abstract

**Background:**

Laryngotracheoesophageal cleft (LTEC) is a rare disease in which the larynx and trachea communicate posteriorly to the esophagus. It is often associated with other congenital malformations, particularly gastrointestinal anomalies. Herein, we report a case of LTEC associated with a gastric polypoid lesion in bronchial tissue.

**Case presentation:**

A gastric mass was detected in a male fetus since week 21 of gestation using fetal ultrasonography. Esophagogastroduodenoscopy performed after birth revealed a pedunculated polypoid lesion of the gastric fornix. The patient experienced frequent vomiting and aspiration pneumonia, which persisted after nasoduodenal tube feeding. Communication between the airway and esophagus was suspected. Laryngoscopy performed 30 days later revealed an LTEC (type III). Partial gastrectomy was performed when the patient was 93 days of age. Histopathological examination revealed tumor consisting of cartilage tissue covered with a layer of respiratory epithelium.

**Conclusion:**

The gastric tumor associated with LTEC exhibited structures mimicking bronchial tissue. LTEC occurs because of foregut maldevelopment, and the tumorous respiratory tissue in the stomach may have been formed from the same abnormal foregut development event as LTEC.

## Background

Laryngotracheoesophageal cleft (LTEC) is a rare congenital anomaly in which the larynx and, in severe cases, the trachea is posteriorly communicated to the esophagus. The abnormality is believed to be caused by failed fusion of the tracheoesophageal septum, which normally begins approximately 4 weeks after conception [1].

Reportedly, LTEC frequently coexists with other congenital malformations. Anomalies of the gastrointestinal system are also frequently observed [1]. Herein, we report a case of a gastric polypoid lesion consisting of bronchial tissue associated with LTEC. Furthermore, we discuss the possible etiology of this unique gastric tumor.

## Case presentation

**Fig. 1:**
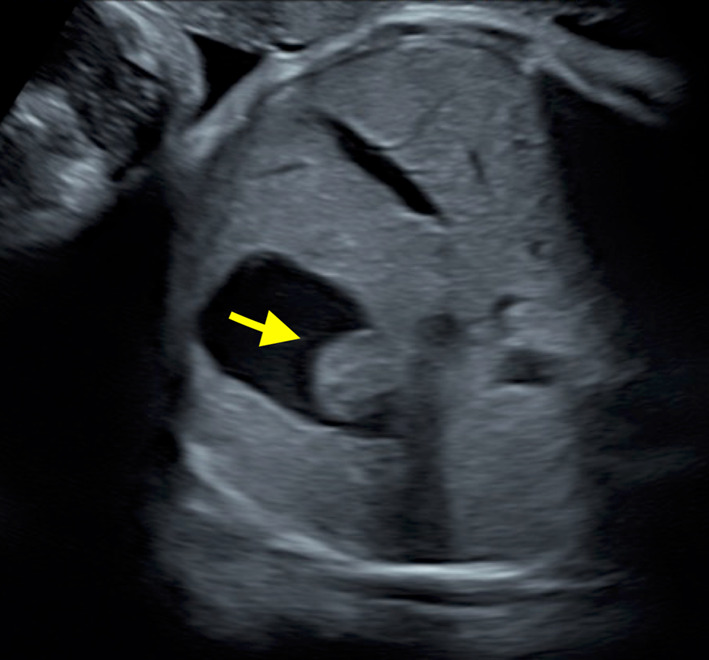
Representative image of a fetal ultrasound study at 21 weeks of gestation. A mass protruding into the lumen of the stomach is observed, demonstrating a mosaic-patterned internal echo (arrow)

**Fig. 2:**
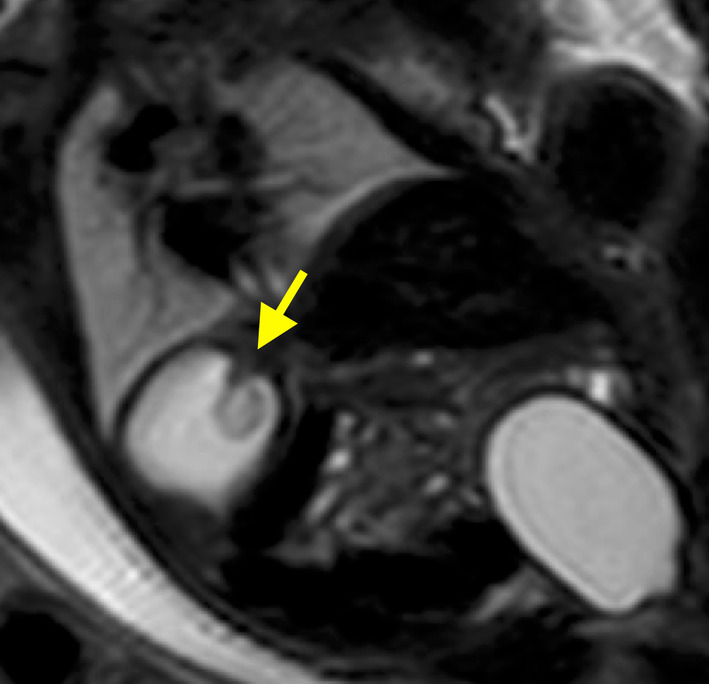
Coronal view of a T2-weighted image from a magnetic resonance imaging study. Obtained at 35 weeks of gestation. A low-intensity polypoid lesion measuring 11 mm in diameter is observed at the gastric fornix (arrow)

A newborn male, weighing 2,377 g, was born at 37 weeks and 3 days of gestation by elective cesarean section and was admitted to the neonatal intensive care unit. A gastric mass with a mosaic-patterned internal echo was detected on fetal ultrasound since week 21 of gestation (Fig. 1). Subsequent fetal magnetic resonance imaging revealed a tumorous lesion measuring 11 mm in diameter at the fornix (Fig. 2). Postnatal abdominal ultrasound confirmed a tumor at the gastric fornix near the gastric cardia measuring 11.4 × 12.4 mm. No cystic or calcified components were identified in the tumor.

**Fig. 3:**
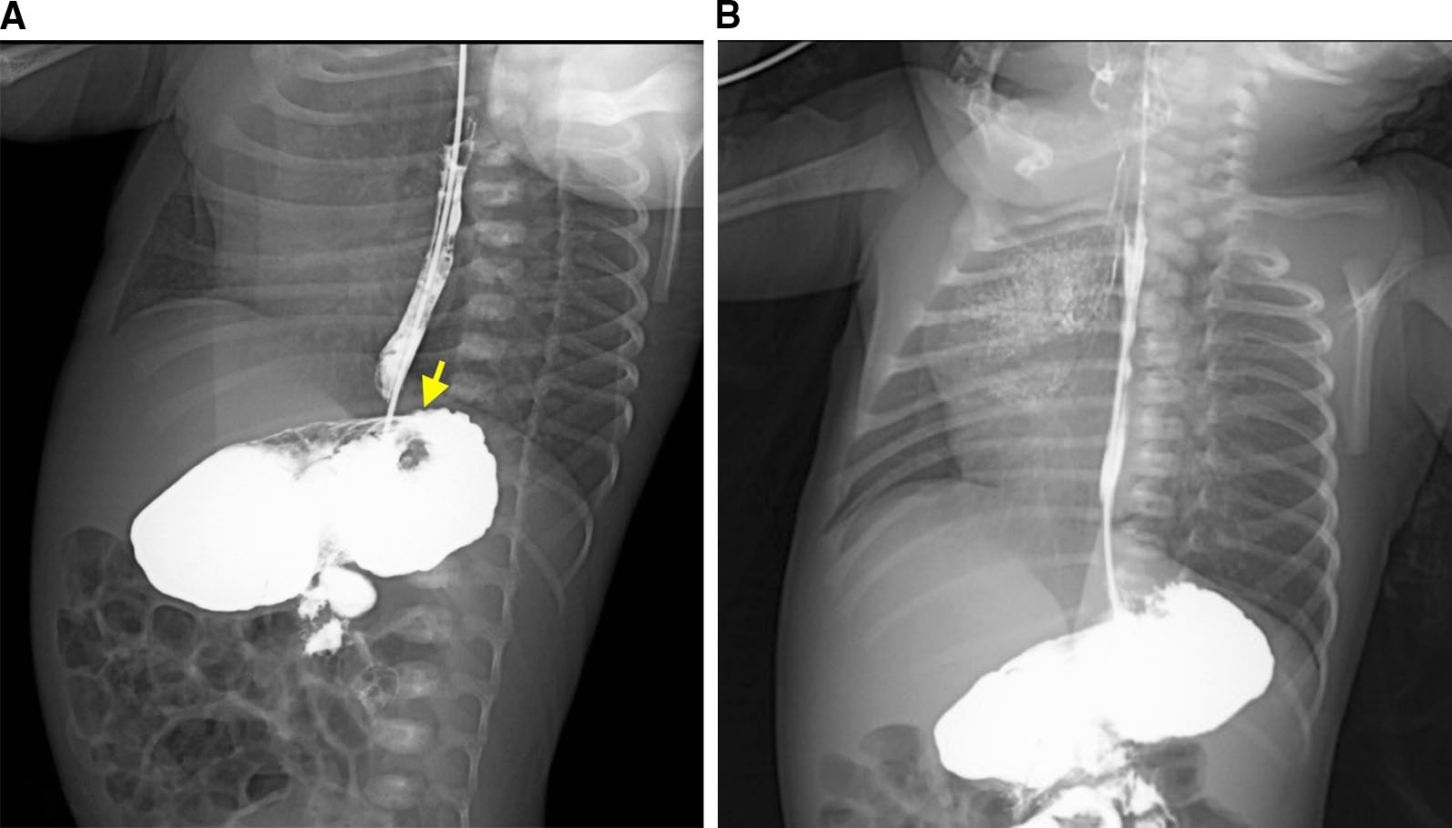
Upper gastrointestinal contrast study at 2 days after birth. **a** The tumor is visualized as a shadow defect (arrow). **b** Severe aspiration of the regurgitated contrast resulted in an extensive bronchogram

**Fig. 4:**
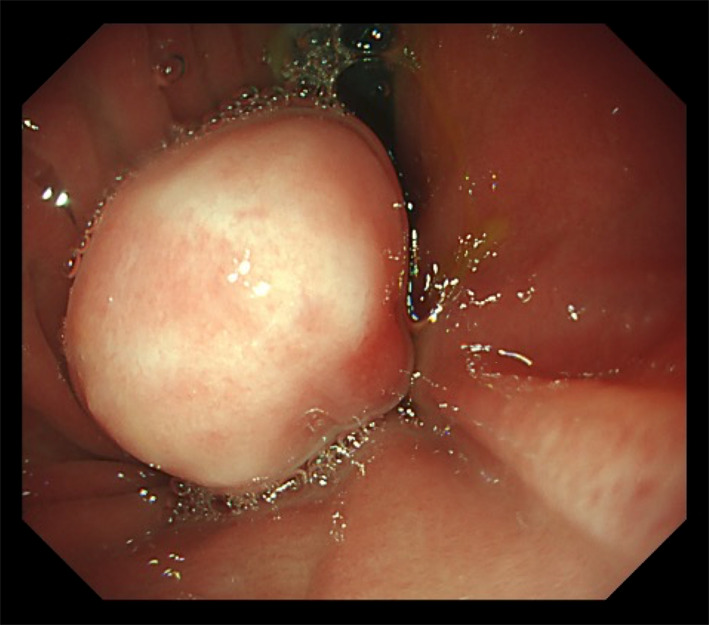
Esophagogastroduodenoscopic findings. An esophagogastroduodenoscopy performed at 23 days of age reveals a tumor at the gastric fornix, 8 mm from the esophagogastric junction. The lesion is pedunculated and covered with a smooth whitish mucosal layer

Frequent vomiting and aspiration were observed during oral feeding. An upper gastrointestinal contrast study performed 2 days after birth revealed a shadow defect in the gastric fornix (Fig. 3A). The angle of His was dull, and severe gastroesophageal reflux was observed. Additionally, aspiration of the regurgitated contrast medium resulted in an extensive bronchogram (Fig. 3B). Esophagogastroduodenoscopy performed at 23 days of age revealed a pedunculated polypoid lesion of the gastric fornix 8 mm from the esophagogastric junction. The lesion was covered with a smooth, whitish mucosal layer (Fig. 4). LTEC was not detected in this series because the patient was intubated.

**Fig. 5:**
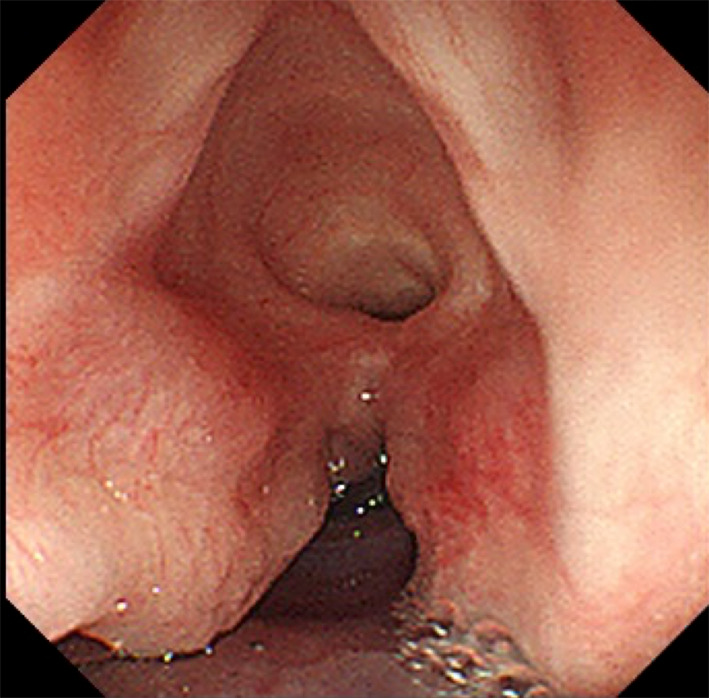
Laryngoscopic findings. Laryngoscopy at 30 days of age reveals a laryngeal cleft continuing beyond the cricoid cartilage but not reaching the first tracheal ring; this led to the diagnosis of laryngotracheoesophageal cleft type III

Vomiting improved after introducing nasoduodenal tube feeding; however, aspiration pneumonia still occurred, and laryngoesophageal or tracheoesophageal communication was strongly suspected. A detailed and thorough inspection using laryngoscopy was performed at 30 days of age to confirm LTEC (type III) (Fig. 5).

**Fig. 6:**
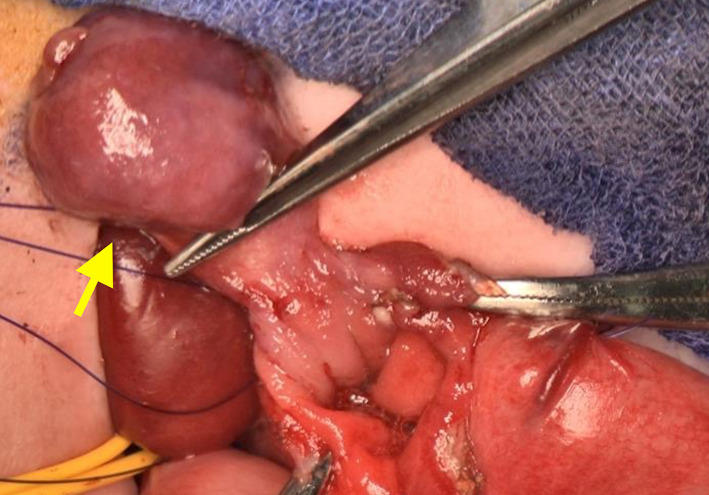
Intraoperative view of partial gastrectomy. The anterior wall of the fornix is opened, and the tumor (arrow) is held with forceps

At 93 days of age, the patient underwent resection of the gastric tumor via partial gastrectomy. The abdomen was opened through a subcostal oblique incision. The tumor was palpable on the posterior wall of the fundus, near the gastric cardia. After mobilizing the gastric fornix, the gastric wall adjacent to the tumor pedicle was incised using an endoscopic view. The tumor was then cored out along the full thickness of the gastric wall to which the pedicle was attached (Fig. 6). The gastric wall defect was repaired, and Toupet fundoplication and gastrostomy were introduced to establish stable enteral feeding. Gastroesophageal reflux was significantly improved thereafter.

A laryngoscopic repair of the LTEC was performed at 198 days of age, thereby successfully preventing aspiration pneumonia. The patient was discharged 315 days of age.

**Fig. 7:**
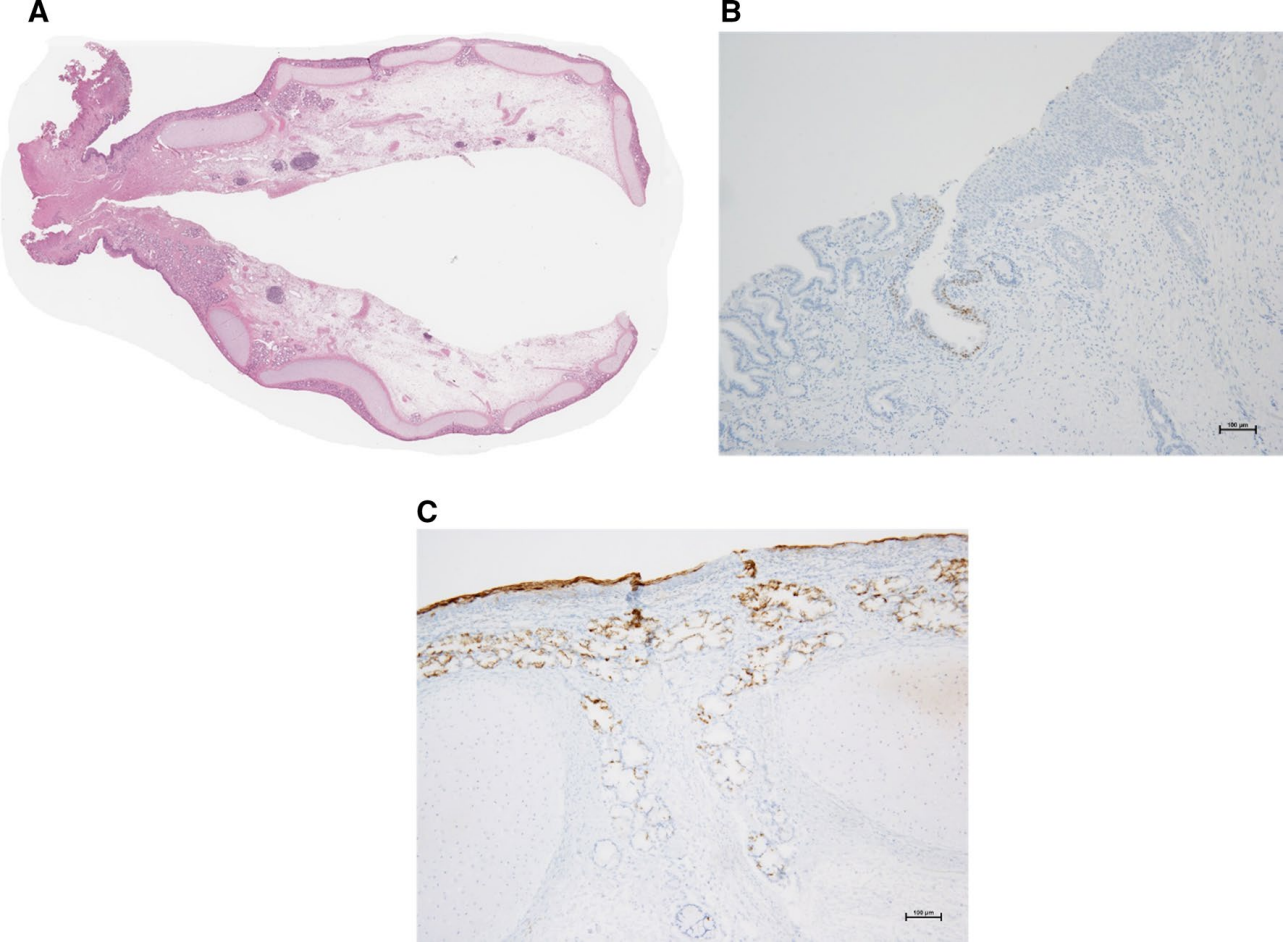
Histological findings of the resected specimen. **a** (10 ×) The epithelium comprises a multilayered columnar epithelium at the pedicle and multilayered squamous epithelium at the apex. A layer of cartilage formed an inverted U-shape in the stroma. Structures mimicking bronchial glands are observed. **b** (100 ×) Immunostaining showed a selected population of cells positive for TTF-1 (shown in brown), a marker for bronchial epithelium, at the epithelia of the pedicle. **c** (100 ×) Diffuse staining of MUC4 (shown in brown) is noted in all layers of the stratified squamous epithelium at the apex, which resembled squamous metaplasia of the bronchial epithelia

The excised gastric polypoid lesion measured 2.7 × 1.6 × 1.5 cm and was lined by a smooth mucosal layer. The cut surface had a solid and myxoid appearance. The epithelium was composed of multilayered columnar epithelium around the pedicle and multilayered squamous epithelium at the apex. The subepithelial stroma was edematous, and structures mimicking bronchial glands were observed. A layer of cartilage formed an inverted U-shape in the stroma, which served as the backbone of the polypoid lesion (Fig. 7A). Immunostaining revealed epithelial cells positive for TTF-1 around the pedicle, which is characteristic of bronchial epithelium (Fig. 7B). At the apex, diffuse staining of MUC4 in all layers of the stratified squamous epithelium was observed (Fig. 7C). The staining pattern of MUC4 was different from that of normal stratified squamous epithelium, in which MUC4 was positive only in the basal cells. The diffuse staining pattern resembled that of squamous metaplasia in the respiratory tract [2].

## Discussion

In the present case, the gastric tumor demonstrated unique histological characteristics resembling bronchial tissue, with a submucosal layer of cartilage covered by a mucosal layer that continued to the gastric mucosa. The mucosa comprised a mixture of stratified columnar epithelium and squamous epithelium, which mimicked bronchial tissue with stratified squamous epithelium metaplasia. Despite a thorough literature search, we could not identify any report describing bronchial or lung tissue forming a solid mass in the stomach, as observed in the present case.

Whether the development of this ectopic bronchial tissue was embryologically in line with the formation of comorbid LTEC is an issue worth discussing. Although a case of ectopic bronchial tissue forming a solid gastric tumor was not found in the literature, cases involving abnormal localization of mature or immature lung tissue within or connecting with the stomach may not be as rare. Bronchopulmonary foregut malformation (BPFM) is defined as the continuity between an isolated piece of respiratory tissue (i.e., the lung, lung lobe, or segment) and the esophagus or stomach [3]. Cases of pulmonary sequestration communicating with the stomach have been previously described [3–6]. Bronchogenic cysts arising from or communicating with the stomach have been reported even more frequently [7]. Furthermore, "foregut duplication cysts" or "foregut cysts" communicating with the stomach, in which the cyst wall is lined with both gastric mucosa and pseudostratified ciliated columnar epithelium, have also been reported [8–11]. Although there are histological variations and various nomenclatures for these anomalies, such abnormalities most likely result from accessory lung buds of the foregut. Gensler et al*.* hypothesized that a gastric foregut cyst mimicking lung tissue was derived from the caudal-most portion of the laryngotracheal outgrowth, which remained attached to the part of the primitive foregut destined to become the stomach [11]. We believe that the gastric bronchial tissue in the present case developed via a similar mechanism.

Meanwhile, LTEC is considered to result from arrests in the separation of the laryngotracheal groove and premature foregut [1]. LTEC is often associated with other disorders originating from abnormal foregut development, including bronchogenic cysts [12], bronchoesophageal fistulas or communicating BPFMs [12, 13], or pulmonary sequestrations [14]. Therefore, we speculate that the LTEC and gastric bronchial tissue originated from a single event during foregut development.

## Conclusions

To the best of our knowledge, this is the first case report to describe a gastric tumor mimicking bronchial tissue associated with LTEC. A gastric lesion associated with LTEC may indicate a lesion related to abnormal foregut development rather than an entirely separate neoplastic lesion.

## Data Availability

All data generated or analyzed during this study are included in this published article.

## Abbreviations

LTEC: Laryngotracheoesophageal cleft
BPFM: Bronchopulmonary foregut malformation
